# Assessing the Influence of Different Comorbidities Indexes on the Outcomes of Allogeneic Hematopoietic Stem Cell Transplantation in a Developing Country

**DOI:** 10.1371/journal.pone.0137390

**Published:** 2015-09-22

**Authors:** Gustavo Machado Teixeira, Henrique Bittencourt, Antonio Vaz de Macedo, Glaucia Helena Martinho, Enrico Antônio Colosimo, Suely Meireles Rezende

**Affiliations:** 1 Post Graduation Program Adult Health, School of Medicine, Universidade Federal de Minas Gerais, Belo Horizonte, Brazil; 2 Hospital das Clinicas, Universidade Federal de Minas Gerais, Belo Horizonte, Brazil; 3 Hematology-Oncology Division, CHU Sainte-Justine, University of Montreal, Montreal, Canada; 4 Department of Pediatrics, School of Medicine, University of Montreal, Montreal, Canada; 5 Institute of Exact Sciences, Department of Statistics, Universidade Federal de Minas Gerais, Belo Horizonte, Brazil; 6 Department of Internal Medicine, School of Medicine, Universidade Federal de Minas Gerais, Belo Horizonte, Brazil; University of Kentucky, UNITED STATES

## Abstract

Although the application of Hematopoietic Cell Transplantation-specific Comorbidity Index (HCT-CI) has enabled better prediction of transplant-related mortality (TRM) in allogeneic hematopoietic stem cell transplants (AHSCT), data from developing countries are scarce. This study prospectively evaluated the HCT-CI and the Adult Comorbidity Evaluation (ACE-27), in its original and in a modified version, as predictors of post-transplant complications in adults undergoing a first related or unrelated AHSCT in Brazil. Both bone marrow (BM) and peripheral blood stem cells (PBSC) as graft sources were included. We analyzed the cumulative incidence of granulocyte and platelet recovery, sinusoidal obstructive syndrome, acute and chronic graft-versus-host disease, relapse and transplant-related mortality, and rates of event-free survival and overall survival. Ninety-nine patients were assessed. Median age was 38 years (18–65 years); HCT-CI ≥ 3 accounted for only 8% of cases; hematologic malignancies comprised 75.8% of the indications for AHSCT. There was no association between the HCT-CI or the original or modified ACE-27 with TRM or any other studied outcomes after AHSCT. These results show that, in the population studied, none of the comorbidity indexes seem to be associated with AHSCT outcomes. A significantly low frequency of high-risk (HCT-CI ≥ 3) in this Brazilian population might justify these results.

## Introduction

Allogeneic hematopoietic stem cell transplantation (AHSCT) is a potentially curative treatment option for different hematological malignancies and non-malignant diseases [1]. Modifications in immunosuppressive therapies, improvements in clinical supportive care, and introduction of reduced-intensity conditioning (RIC) regimens have led to an increased indication of transplant to patients who were otherwise not previously eligible for AHSCT with a myeloablative conditioning regimen (MA), such as the elderly [2]. Nevertheless, AHSCT continues to have a significant morbimortality rate [3].

Three main factors influence mortality and survival rates after AHSCT: the primary disease *per se*, donor type, and patient-related factors, such as the presence of comorbidities [2].

Comorbidity indexes have been developed to evaluate the impact of these comorbidities on different clinical situations, including cancer and HSCT [4–7] Charlson’s Comorbidity Index (CCI) and the Adult Comorbidity Evaluation (ACE-27) are valuable tools in predicting mortality in cancer patients [4,6] However, CCI has shown a low sensitivity for this purpose in AHSCT [7,8]. In an attempt to improve the assessment of the comorbidity risk profile in AHSCT patients, the Hematopoietic Cell Transplantation-specific Comorbidity Index (HCT-CI) was developed [7,9] The HCT-CI included a larger number of pre-transplant comorbidities compared to CCI and provided a better predictability of transplant-related mortality (TRM) and overall survival (OS) [7,9]. The HCT-CI has been validated in several developed countries as a TRM and OS predictor [9–15] However, not all studies have confirmed its predictive value for TRM [16–18].

In developing countries, data on the impact of comorbidities on AHSCT outcomes remain scarce. Three studies investigated the incidence of comorbidities and their effects on transplant-related mortality in developing countries [12,19,20]. However, these studies had retrospective design and they did not draw any definitive conclusion. Therefore, we aimed at evaluating, in a prospective study, the HCT-CI and the ACE-27 indexes as predictors of AHSCT complications in a Brazilian HSCT unit.

## Materials and Methods

### Study

A prospective cohort study was conducted in the HSCT Unit of Hospital das Clínicas at Universidade Federal de Minas Gerais (UFMG), Belo Horizonte, Brazil. The inclusion period ranged from March 1^st^, 2008 to March 31^st^, 2013. The study was approved by the institutional Ethics Committee, and all patients included signed an informed consent form.

### Patients

Patients with hematologic malignancies and non-malignant diseases, aged ≥ 18 years, and undergoing a first related or unrelated AHSCT were considered eligible. Transplants using umbilical cord blood (UCBHSC) as graft source (n = 1 during the study period) were excluded.

### Variables

Primary diseases included hematologic malignancies (acute leukemia, chronic myeloid leukemia, multiple myeloma, primary myelofibrosis, lymphomas, myelodysplastic syndrome, and unspecified myeloproliferative disorders) and non-malignant diseases (aplastic anemia, paroxysmal nocturnal hemoglobinuria, Fanconi anemia, and congenital dyskeratosis). For malignant disorders, a modification of the neoplastic disease /disease status index (DR/DS) was used [21], which allowed for stratification of the hematologic malignancies into two categories: low/intermediate and high/very high risk.

Information on patients’ comorbidities was obtained by direct interviews with the patient and/or corresponding family members, and by consulting laboratory, radiological, echocardiogram, electrocardiogram and pulmonary function tests that had constituted the pre-AHSCT work-up. Furthermore, additional data were obtained from medical records, when needed. Patients were stratified according to their scores on the HCT-CI [7] and ACE-27 [6]. The next step was to create a modified version of the original ACE-27 by excluding hematologic malignancies as a classification criterion.

AHSCT was defined as “related” when the donor comprised a family member, and “unrelated”, when the donor was a volunteer enrolled in national or international registries. Graft source included: bone marrow hematopoietic stem cells (BMHSC) and peripheral blood hematopoietic stem cell (PBHSC).

Conditioning regimens were classified according to the definitions from the National Marrow Donor Program and the Center for International Blood and Marrow Transplant Research [22, 23] and grouped into: (i) myeloablative conditioning regimens (MA), when a total busulfan (BU) (oral formulation) dose of ≥ 9 mg/kg and/or intravenous melphalan (MEL) dose of ≥ 140 mg/m^2^ were used; (ii) Reduced intensity regimens (RIC) included busulfan-melphalan, busulfan-fludarabine, cyclophosphamide alone, and fludarabine-cyclophosphamide combinations.

Alemtuzumab, combined with the conditioning regimen, was used in all unrelated AHSCT patients and in related AHSCT cases of polytransfused aplastic anemia (≥ 10 prior blood transfusions), as well as for patients aged ≥ 45 years with myelodysplasia, acute leukemia, chronic myeloid leukemia or myelofibrosis.

### Outcomes

Outcomes evaluating recovery of hematopoiesis were granulocyte recovery (GR) and platelet recovery (PR), defined as the first of three consecutive days with neutrophil counts above 500 cells/mm^3^ and the first of seven consecutive days with platelet counts ≥ 20,000/mm^3^ with no need for platelet transfusion, respectively. Diagnosis of sinusoidal obstructive syndrome (SOS) was based on the clinical criteria proposed by the Seattle group [24] Diagnosis and classification of acute graft-versus-host disease (GVHD) were based on the criteria proposed by Glucksberg et al. For this analysis, only acute GVHD grade II to IV were considered [25]. For chronic GVHD, diagnosis was based on the criteria proposed by the National Institutes of Health [26].

Relapse was considered as follows: (i) in acute leukemia, presence of > 5% blasts in bone marrow aspirate and/or immunophenotyping; (ii) in lymphomas, recurrence of lymphadenopathy and/or lymphoid organ infiltration, with a histopathological confirmatory diagnosis; (iii) in myelofibrosis, resurgence of hepatosplenomegaly, and bone marrow trephine biopsy showing fibrosis, and (iv) in chronic myeloid leukemia, molecular relapse through presence of increasing levels of the BCR-ABL fusion gene by quantitative real time PCR. Cytogenetic relapse was defined by the reappearance of t(9;22) in post-transplant cytogenetic assessments.

Transplant-related mortality (TRM) was defined as the cumulative incidence (CI) of death associated with AHSCT complications rather than recurrence of primary disease within a period of two years after AHSCT. Event-free survival (EFS) was the probability of being alive and without relapse within two years after transplant, whereas OS was the probability of being alive in the first two years after AHSCT.

### Statistical analysis

Description of variables included frequency (n) and percentages, for categorical variables, and median, minimum and maximum values, for continuous variables. EFS and OS probabilities were calculated using the Kaplan-Meier method and the log-rank test for comparisons between groups. The data were censored at the time of death or up to the last contact date according to medical records. We used Gray’s model in the analysis of incidence of competing events, whereby death was considered as a competitive event in the analysis of platelet and granulocyte recovery, SOS development, acute and chronic GVHD, and relapse. In the analysis of the cumulative incidence of TRM, relapse was considered as a competing event. The hazard ratio (HR) was estimated with a confidence interval of 95% (CI 95%). The multivariate analysis of EFS and OS used Cox’s regression model, while the multivariate analysis of competing events for other outcomes was performed using the Fine and Gray method. The following variables were used in the univariate analysis: HCT-CI, ACE-27, modified ACE-27, primary disease, donor type, graft source, conditioning regimen, and use of alemtuzumab. In the assessment of relapse, the DR/DS index was also used. In multivariate analysis, the HCT-CI and the ACE-27 and modified ACE-27 indexes were included, as well as the variables that had presented a p value ≤ 0.3 in univariate analysis. All statistical models were analyzed using the Easy R software [27].

## Results

### Patients

A total of 108 adult patients underwent an AHSCT during the study’s recruitment period. Of these, 9 patients did not consent to participate. Thus, 99 patients were included in the study. Males comprised 60.6% (n = 60). The median age at transplantation was 38 years (range, 18 to 65 years), and patients aged ≥ 50 years represented 22.3% of the total. Hematologic malignancies accounted for 75.8% of all cases (n = 75). Other demographic characteristics are shown in Table 1.

**Table 1:** Clinical characteristics of the patients.

Characteristics	Data	
Sex, male (%)	60	(60.6)
Age at transplant, median in years (range)	38	(18–65)
Hematological malignancy, n (%)	75	(75.8)
**Diagnosis, n (%)**		
Acute myeloid leukemia	30	(30.3)
Aplastic anemia	20	(20.2)
Acute lymphoblastic leukemia	12	(12.1)
Chronic myeloid leukemia	10	(10.1)
Non-Hodgkin lymphoma	8	(8.1)
Myelodisplastic syndrome	7	(7.1)
Other*	12	(12.1)
**Neoplastic disease/disease status index** ** **n (%)**		
Low/intermediate	46	(61.3)
High/very high	29	(38.7)
**Donor, n (%)**		
Related/unrelated	88/11	(88.9/11.1)
**Conditioning regimen, n (%)**		
Myeloblative/reduced-intensity conditioning	55/44	(55.6/44.4)
**Use of alemtuzumab, n (%)**	49	(49.5)
**Graft source, n (%)**		
Bone Marrow/peripheral blood	27/72	(27.3/72.7)
**HCT-CI, n (%)**		
HCT-CI: 0	62	(62.6)
HCT-CI: 1–2	29	(29.3)
HCT-CI: ≥3	8	(8.1)
**ACE-27, n (%)**		
ACE-27 = 0	17	(17.2)
ACE-27 = 1	20	(20.2)
ACE-27 = 2	19	(19.2)
ACE-27 = 3	43	(43.4)
**Modified ACE-27** *** **, n (%)**		
Modified ACE-27 = 0	53	(53.5)
Modified ACE-27 = 1	35	(35.4)
Modified ACE-27 = 2	9	(9.1)
Modified ACE-27 = 3	2	(2.0)

**Abbreviations: ACE-27,** Adult Comorbidity Evaluation; **HCT-CI**, Hematopoietic Cell Transplantation-specific Comorbidity Index

*Other: includes Fanconi anemia, congenital dyskeratosis, paroxysmal nocturnal hemoglobinuria, multiple myeloma

** Adapted from Armand *et al* [21]

*** Excluding hematologic malignancies.

### Recovery of hematopoiesis

Cumulative incidence of granulocyte recovery (GR) at 30 days was 84.8%, with a median time of 19 days (range, 9 to 35 days). The cumulative incidence of platelet recovery (PR) was 80.8% at 180 days, with a median time of 20 days (range, 9 to 110 days). None of the comorbidity indexes, HCT-CI, ACE-27 and modified ACE-27, influenced GR or PR (Table 2).

**Table 2:** Results of multivariate analysis.

Outcomes	Variables included in the model	Significant variables
**Granulocyte recovery**	HCT-CI, ACE-27, modified ACE-27 and graft source	None
**Platelet recovery**	HCT-CI, ACE-27, modified ACE-27 and donor type	None
**SOS**	HCT-CI, ACE-27, modified ACE-27, primary disease and conditioning regimen	None
**Acute GVHD**	HCT-CI, ACE-27, modified ACE-27, primary disease, conditioning regimen and alemtuzumab	None
**Chronic GVHD**	HCT-CI, ACE-27, modified ACE-27, primary disease, donor type, conditioning regimen and alemtuzumab	RIC: HR = 0.17 (0.05–0.53;p = 0.002)
**Relapse**	HCT-CI, ACE-27, modified ACE-27, graft source, RD/DS and donor type	High/very high DR/DS: HR = 2.18 (1.06–4.48; p = 0.03)
**TRM**	HCT-CI, ACE-27, modified ACE-27, primary disease and donor type	None
**EFS**	HCT-CI, ACE-27, modified ACE-27, primary disease, graft source, conditioning regimen and alemtuzumab	Primary disease (Neoplasia): HR = 3.68 (1.43–9.47; p = 0.006)
**OS**	HCT-CI, ACE-27, modified ACE-27, primary disease, graft source, conditioning regimen and alemtuzumab	None

**Abbreviations: ACE-27 =** Adult Comorbidity Evaluation; **DR/DS =** Neoplastic disease/disease status index; **EFS** = Event-free survival; **GVHD =** Graft-versus-host disease; **HR** = Hazard ratio; **HCT-CI** = Hematopoietic Cell Transplantation-specific Comorbidity Index; **OS** = Overall survival; **RIC =** Reduced-intensity conditioning; **SOS** = Sinusoidal obstructive syndrome; T**RM** = Transplant-related mortality

### Sinusoidal obstructive syndrome

The cumulative incidence of SOS at 30 days was 18.2%, with a median time of 10.5 days (range, 0 to 17 days). In the univariate analysis, only the presence of a hematologic malignancy (22.7% vs. 4.2%, p = 0.04) and use of a MA regimen (25.5% vs 9.1%, p = 0.04) were associated with an increase in the incidence of SOS. Multivariate analysis did not confirm the influence of these variables on the incidence of SOS. Similarly, the HCT-CI, ACE-27 and modified ACE-27 did not affect the incidence of SOS (Table 2).

### Graft-versus-host disease

The cumulative incidence of acute grade II-IV GVHD at 100 days was 28.3%, with a median time of 23 days (range, 12 to 100 days). In the univariate analysis, only the presence of a hematologic malignancy (34.7% versus 8.3%, p = 0.01) and a higher ACE-27 index (7.6%, 5.0%, 36.8% and 39.5% for an ACE-27 index of 1, 2, 3 or 4, respectively, p = 0.02) showed an association with the incidence of acute GVHD. In the multivariate analysis, the HCT-CI, ACE-27, modified ACE-27, hematologic malignancy, use of alemtuzumab, and unrelated AHSCT were not risk factors for the development of acute GVHD (Table 2).

The cumulative incidence of chronic GVHD at two years was 31.4% in 70 patients at risk, with a median time to onset of 179 days (range, 59 to 351 days). In the univariate analysis, ACE-27 (p = 0.004), graft source (p = 0.05), primary disease (p = 0.01) and type of conditioning regimen (p <0.001) were associated with chronic GVHD. Neither the HCT-CI or the ACE-27 showed any association with the incidence of chronic GVHD in multivariate analysis. Use of RIC was the only variable associated with a lower incidence of chronic GVHD (HR = 0.17; 95% CI, 0.05 to 0.53; p = 0.002).

### Relapse

The cumulative incidence of relapse at two years was 37.9%, with a median time of 0.4 year (range, 0.1 to 1.9 years). In the univariate analysis, only high/very high DR/DS (50.1% versus 30.4%; p = 0.02) was associated with incidence of relapse. This was confirmed in the multivariate model (HR = 2.18; 95% CI = 1.06–4.48; p = 0.03). None of the three comorbidity indexes studied had any influence on the relapse rate (Table 2).

### Transplant-related mortality

The cumulative incidence of TRM at two years was 37.4%, with a median of 0.6 year (range, 0 to 2 years). Causes of death were infection (n = 23), SOS (n = 6) and acute GVHD (n = 7). Through univariate analysis, only the ACE-27 index was associated with TRM, where score 0, 1, 2 and 3 presented an incidence of 29.4%, 30.0%, 15.5% and 48.8%, respectively (p = 0.04). In the multivariate analysis, none of the studied variables were predictive for TRM (Table 2).

### Event-free and overall survival

EFS at two years was 43.2%, with a median time of 0.6 year (range, 0.2 to 5.5 years). In univariate analysis, type of primary disease (33.0% for hematologic malignancies versus 75.0% for non-malignant diseases, p < 0.001), graft source (37.1% for PBHSCT versus 59.3% for BMHSCT; p = 0.03), type of conditioning regimen (34.0% for MA versus 50.0% for RIC; p = 0.02) and ACE-27 (64.7%, 65.0%, 52.6% and 20.3% for score 0, 1, 2 and 3, respectively; p < 0.001) influenced EFS. In the multivariate analysis, only the presence of a hematologic malignancy was predictive for EFS (HR = 3.68; 95% CI = 1.43–9.47; p = 0.006). None of the comorbidity indexes influenced EFS in the multivariate analysis (Table 2).

The OS at two years was 41.3%, with a median of 0.9 year (range, 0.5 to 5.5 years). In the univariate analysis, primary disease (p = 0.001) and ACE-27 (p < 0.001) influenced OS, which was not confirmed in multivariate analysis (Table 2).

## Discussion

This study evaluated the presence and relevance of comorbidities as predictors for complications related to AHSCT using HCT-CI, ACE-27, and modified ACE-27 indexes. We did not show any association between these comorbidity indexes and any of the post-transplant clinical outcomes studied.

Sorror *et al* [7], in a large retrospective single-center study of 1,055 patients, and Raimondi *et al* [13], in a prospective multi-center study with 1,937 patients, confirmed the predictive value of the HCT-CI on TRM and OS. Both studies included patients with malignant and non-malignant hematologic diseases who underwent related or unrelated HSCT, and neither of them included UCBHSC as graft source. Unlike these studies, we could not confirm this predictive value for TRM and OS. Nevertheless, there are important differences between our study population and those of the above mentioned studies, such as: (i) a younger population in our study (median age of 38 years compared to 44.8 years and 47 years in the studies by Sorror *et al* and Raimondi *et al*, respectively); (ii) a significantly lower proportion of patients classified as HCT-CI ≥ 3 in our study (8% compared to 28% in Sorror *et al* and 19% in Raimondi *et al*); (iii) a higher proportion of non-malignant hematologic diseases in our study (24.0% versus 3.0% in Sorror’s study and 4.9% in Raimondi’s study) and (iv) a significantly lower proportion of unrelated AHSCT in our study (11%), since, in the studies by Sorror *et al*, and Raimondi *et al*., these accounted for 42% and 50.5% of cases, respectively [7,13].

Likewise, a few other studies did not confirm the predictive value of the HCT-CI on AHSCT clinical outcomes [16,18]. Birninger *et al*, in a retrospective single-center study of 340 patients (adults and children) with acute myeloid leukemia, did not report any influence of the HCT-CI on TRM and OS [16]. However, when comparing the Birninger study population and that of the present study, major differences were found, especially regarding the proportion of hematologic malignancy (100% versus 75.8%), age (median 53 versus 38 years), and the percentage of patients with HCT-CI ≥3 (74% vs 8%), respectively. In another single-center prospective study of 187 patients undergoing related and unrelated AHSCT for hematologic diseases, the predictive value of the HCT-CI for TRM and OS was also not confirmed [18]. Some of the characteristics of this study were similar to ours, such as median age (39 years) and proportion of patients with hematologic malignancy (79%). However, there was a greater use of BMHSC (72.0% versus 27.3%) and a larger proportion of patients with HCT-CI ≥ 3 (55% *versus* 8%) in the study by Guilfoyle *et al* [18] when compared to our study.

With regard to other outcomes, a retrospective study of 2,985 patients who had undergone related or unrelated AHSCT showed that the HCT-CI was an independent risk factor for the development of acute GVHD, especially of its severe forms (grade III-IV) [28]. In our study, we did not find an association between the HCT-CI and the incidence of acute GVHD (II-IV), although there are differences between the population in our study when compared to that of Sorror *et al* [18], such as a lower proportion of patients aged ≥ 50 years (22.3% versus 38.0%), lower proportion of unrelated AHSCT (11% versus 45%), and lower proportion of patients classified as HCT-CI ≥ 3 (8% versus 37%), respectively. It is noteworthy, however, that we did not evaluate the association between the HCT-CI and the severity of acute GVHD, because of the small number of patients who developed severe forms (n = 7). Sorror’s study did not use alemtuzumab, whereas the use of this medication in a significant number of patients in our study may have contributed to the reduced incidence of severe forms of acute GVHD observed.

ACE-27, a comorbidity index developed for cancer patients, did not show to be a good predictor of transplant-related complications, since it includes hematologic malignancies among its scoring criteria. Even the modified ACE-27 index, which excludes hematologic malignancies from its criteria, did not prove to be of much benefit when used in the AHSCT setting, as it contains comorbidities that are rarely found in patients who are candidates for this therapeutic modality, such as acquired immunodeficiency syndrome, congestive heart failure, and severe dementia.

The use of RIC regimens in our study was associated with a significant reduction in the incidence of chronic GVHD amongst patients at risk for this complication. This difference is also likely a result of a significant number of patients who were transplanted with a non-malignant disease, an increased use of alemtuzumab, and the use of BMHSC as graft source in the RIC group. Potter *et al*. and Marsh *et al*. reported that the use of alemtuzumab in RIC regimens results in a low incidence of chronic GVHD [29,30]. As to the type of graft source, two meta-analysis studies have suggested that use of PBHSC as a cell source is a risk factor for the development of chronic GVHD, when compared to the use of bone marrow [31,32].

The DR/DS index, as expected, was associated with the incidence of relapse, where patients classified as high/very high risk showed a high incidence of this outcome, which is in accordance with the previous publication by Armand *et al* [21].

There are some important limitations of this study which are worth mentioning, such as its single-center nature and the small number of patients who were classified as HCT-CI ≥ 3. The lower proportion of patients stratified as HCT-CI ≥ 3 is probably a result of the use of stricter selection criteria for the indication of this procedure by the transplant team and/or the fact that, in Brazil, there is still a waiting list for AHSCT, which may account for the selection of “fitter” patients for transplant. On the other hand, our study is highly significant in that it has a prospective design and targets a particularly underrepresented population in the literature: that of the developing countries.

## Conclusion

We have shown that in the population studied, we did not confirm the predictive value of the HCT-CI for the different AHSCT-related outcomes. Furthermore, the original ACE-27 and its modified index are not adequate tools for use in the AHSCT setting, mainly because they either include hematologic malignancies in their criteria or encompass comorbidities that are rarely found in transplant candidates.
